# Total knee arthroplasty in carefully selected patients aged 80 years or older

**DOI:** 10.1186/s13018-014-0061-z

**Published:** 2014-07-15

**Authors:** Feng-Chih Kuo, Chi-Hsiang Hsu, Wun-Schen Chen, Jun-Wen Wang

**Affiliations:** 1 Department of Orthopedic Surgery, Kaohsiung Chang Gung Memorial Hospital, Chang Gung University, College of Medicine, No.123, Ta Pei Road, Niao Sung Dist, Kaohsiung City 833, Taiwan

**Keywords:** Octogenarian, Total knee arthroplasty, Mortality, Postoperative complications

## Abstract

**Background:**

The patients aged ≥80 years have been considered to have a higher risk of mortality, postoperative complications, and longer hospital stay following total knee arthroplasty (TKA) than younger patients. The purposes of this retrospective study were to review the results of TKA in patients aged ≥80 years after a preoperative consultation.

**Methods:**

Seventy-five patients aged ≥80 years underwent TKA from January 2006 and June 2010. A control group of younger patients (65–74 years) was matched in a 1:1 ratio with the ≥80 years group for sex, diagnosis of the disease, body mass index, the American Society of Anesthesiologists' type of anesthesia, and comorbidities. Cardiologists and neurologists carefully evaluated the risk of patients for both groups before surgery. The groups were compared with regard to Knee Society Scores, Knee Society Function Score, Western Ontario and McMaster Universities Osteoarthritis Index scores, length of stay, postoperative complications, and 90-day mortality rate.

**Results:**

The mean follow-up was 2.3 years (range 1–5 years). We found no difference in the functional outcomes and length of stay between the two groups. The ≥80 years group had a higher rate of blood transfusion (29.3% versus 10.7%, *p* = 0.006) after Bonferroni correction. There were no cardiovascular or cerebrovascular complications in the ≥80 years group. There were no mortalities within 90 days in either group.

**Conclusions:**

Despite similar functional results and pain relief of the TKA compared with the young patient group, the ≥80 years group had a higher complication rate of blood transfusion. With a preoperative consultation by cardiologists and neurologists, patients aged ≥80 years have a low cardiovascular or cerebrovascular complications and 90-day mortality after TKA.

## Background

Total knee arthroplasty (TKA) is an effective surgical intervention, which alleviates pain and improves function and health-related quality of life in patients with late-stage arthritis of the knee joint [1],[2]. With improvements in anesthesia, general health care, and surgical techniques, this procedure has become widely accepted for use in very elderly patients, including those more than 80 years old. However, a recent study showed that despite similar pain relief and functional improvement, compared with a younger group of patients, octogenarian had a higher risk of death, postoperative complications and longer hospital stay after standard TKA [3].

The purpose of this retrospective study was to compare clinical functional outcomes, postoperative complications, length of stay, and 90-day mortality after TKA between patients aged ≥80 years and a younger patient group.

## Methods

We performed a retrospective case-controlled study with a one-to-one ratio of cases and matched controls. Using our institutional total joint registry database, we identified all patients who were aged 80 years or older undergoing primary total knee arthroplasty at our institution from January 2006 and June 2010. One thousand twenty-four patient (1,186 TKAs) total knee arthroplasty procedures were performed during the study period.

We designed a standard protocol for patients aged ≥65 years before they underwent major orthopedic surgery including TKA at our institution. Routine preoperative consultation with cardiologists and neurologists were conducted for these patients who were willing to undergo a TKA. The cardiologist evaluated these patients based on the American Heart Association (AHA) guidelines on perioperative cardiovascular evaluation and care for patients undergoing noncardiac surgery [4]. The cardiologist would use these interventions, including a 12-lead electrocardiogram (ECG), exercise stress test, noninvasive stress test (thallium-201 myocardial perfusion scintigraphy), or echocardiography to predict perioperative cardiovascular risk. If the perioperative cardiovascular risk was high (>5%), surgery was not recommended. If the perioperative cardiovascular risk was low (<5%), the patient was going to have surgery. The neurologist used duplex ultrasounds to evaluate the carotid and vertebrobasilar artery stenosis to predict the risk of perioperative stroke [5],[6]. Patients with symptomatic carotid bruits or a history of stroke or transient ischemic attack and a documented stenosis of >50% may have a risk for stroke as high as 3.6% [7]. Therefore, surgery was not recommended. During the study period, 103 patients aged 80 years or older were referred to cardiologists and neurologists for preoperative consultation. Twenty-eight of these patients (27%) were not considered for surgery because of high risk of perioperative cardiovascular and cerebrovascular disease. Therefore, 75 patients aged 80 years or older were enrolled to the study. For each patient aged 80 years or older, we aimed to identify one matching patient aged between 65 and 74 years as a control. This control population was derived with use of our electronic database by matching for the following variables: sex, diagnosis of the disease, body mass index (BMI), American Society of Anesthesiologists (ASA), type of anesthesia (regional or general), and comorbidities. Matched variables were chosen to eliminate their potential confounding effects on study outcomes. The exclusion criteria were bilateral total knee arthroplasty and less than 6 months of follow-up after the procedure of interest. This study was approved by the institutional review board of Chang Gung Memorial Hospital (IRB No.: 101-3512B).

Demographic data, including age, sex, BMI, knee disease (osteoarthritis, rheumatoid arthritis, or post-traumatic arthritis), ASA, type of anesthesia (general or spinal), preoperative hemoglobin (Hb) and hematocrit (Hct) levels, total 48-h postoperative tube drainage, wound length, and length of stay, were recorded. Other preoperative comorbidities were also recorded: diabetes mellitus, cardiac disease (hypertension, CAD, congestive heart failure, and arrhythmia), renal insufficiency, liver cirrhosis, chronic obstructive pulmonary disease (COPD), adrenocortical insufficiency, peptic ulcer, gout, and coagulopathy.

### Surgical technique

All operations were performed or supervised by the senior author (JWW) and employed the mini-midvastus approach for TKA, as described by Haas et al.: ‘The skin incision was made along the medial aspect of the patella to the medial border of the mid-to-distal tibial tubercle [8]. The vastus medialis oblique muscle was split approximately 2 cm in line with its fibers from the superior medial pole of the patella.’ All TKAs were unilateral and cemented, and the same prosthesis was used (Nex-Gen, Legacy Posterior Stabilized Prosthesis; Zimmer, Warsaw, IN, USA). We used an intramedullary alignment rod for femoral cutting and an extramedullary guide system for tibial cutting. The femoral canal for intramedullary guiding was routinely plugged with bone. Meticulous electric cauterization of the soft tissue bleeding points was performed throughout the surgery. The tourniquet was not released until skin closure and application of a compressive dressing. Intraoperative blood loss was negligible in all patients because the tourniquet was not deflated until wound closure. A drain was put in the knee joint before wound closure.

### Postoperative care

A standard postoperative rehabilitation protocol, including continuous passive motion of the knee and muscle strengthening exercise immediately after returning to the ward, was followed in both groups. All patients were asked to get out of bed with walker support on the afternoon of the first postoperative day to prevent deep-vein thrombosis (DVT), as described by Pearse [9]. We subcutaneously administered 20 mg enoxaparin (Clexane; Glaxo-Smith-Kline, Brentford, Middlesex, UK) in all patients as postoperative prophylaxis for DVT every 12 h until discharge. Then, they received indomethacin (orally or by suppository) for at least 4 weeks [10].

All patients returned to the clinic 2 weeks after surgery for suture removal and clinical examination, at 6 weeks for clinical evaluation of functional recovery, and at 12 weeks for radiographic examination of the knee. Partial weight bearing with a cane support usually was allowed 4–6 weeks after surgery, and full weight bearing without a support usually was allowed 6–12 weeks after surgery.

We applied the principle of the criteria and guidelines for perioperative transfusion suggested by the National Institutes of Health Consensus Conference, which state that the decision to transfuse blood depends on clinical assessment, aided by laboratory data, relevant to symptoms and signs of acute anemia [11].

DVT was suspected in the presence of swelling or edema of the thigh or calf, greater than 3 cm, compared to the contralateral leg with calf tenderness for as long as 12 weeks after the operation. We did not routinely screen for venous thrombosis, but all clinically suspected thrombosis or pulmonary embolisms (PEs) were investigated using antegrade venography or CT of the chest.

Patients were assessed using the Knee Society Scores (KSS), the Knee Society Function Score (KSFS), the Western Ontario and McMaster Universities Osteoarthritis Index (WOMAC) scores, and range of knee motion (ROM) before operation and at the latest follow-up. Radiographic evaluation included standing anteroposterior radiographs of the knee and a long weight-bearing film of the limb to measure the tibiofemoral mechanical axis (TFM) before operation and at the latest follow-up. A postoperative outlier was defined if there was >3° deviation from the ideal (TFM = 0°). We obtained data regarding medical complications (confusion, pneumonia, myocardial infarction [MI], DVT with positive venogram finding, transfer to intensive care unit, postoperative congestive heart failure [CHF], pulmonary embolism, upper gastrointestinal [UGI] bleeding, cerebral vascular accident/transient ischemic accident (CVA/TIA), postoperative anemia, and atelectasis) and surgical complications (hematoma around the knee, superficial wound infection) within 90 days after operation. We also ascertained the 90-day postoperative mortality. Other complications, including periprosthetic fractures around the knee, deep infection or loosening of the implant were recorded.

### Statistical analysis

All statistical comparisons were made using Statistical Package for Social Sciences (SPSS) software (version 15; Chicago, IL, USA). A sample size of 150 patients achieves 85% power to detect an effect size (W) of 0.2513 using a 1 degree of freedom chi-square test with a significance level (alpha) of 0.05000. We compared differences in age, BMI, wound length, length of stay, and preoperative Hb and Hct levels, tube drainage, mechanical axis, range of motion, and function scores (the KSS, the KSFS, and the WOMAC score) between two groups using independent *t* test. We compared difference in diagnosis, anesthesia type, ASA, preoperative comorbidity and postoperative complications using chi-square test or Fisher's exact test. A Bonferroni-adjusted significance level of 0.00416 was calculated to account for the increased possibility of a type-I error.

## Results

The mean duration of follow-up was 2.3 years (range 1–5 years). The mean age at operation was 82 years (range 80–93 years) among the ≥80 years group and 72 years (range 65–74 years) among the controls. The two patient groups were similar with respect to sex, BMI, diagnosis, ASA, type of anesthesia (Table 1), mean preoperative KSS, KSFS, WOMAC score, preoperative ROM and knee alignment (TFM) (Table 2), and comorbidities (Table 3).

**Table 1:** Patients' characteristics

**Parameters**	**≥80 years group**	**Control group**	***p*** **value**
Mean age (year)	82 (2.8; 80–93)	72 (2.2; 65 ~ 74)	<0.001
Female, *n* (%)	54 (72)	62 (82)	0.144
Mean BMI (kg/m^2^)	26.2 (3.0; 19.5–35.5)	28.2 (3.7; 21.5–38.9)	0.078
OA/RA/PTA, *n*	73/2/0	72/2/1	0.528
ASA risk, *n* (%)			0.1
I	0	2 (2.7)	
II	42 (56)	50 (66.6)	
III	33 (44)	23 (30.7)	
Anesthesia, *n* (%)			0.684
General	73 (97.3)	72 (96)	
Spinal	2 (2.7)	3 (4)	

Numbers in parenthesis are standard deviation and ranges. *OA* osteoarthritis, *RA* rheumatoid arthritis, *PTA* post-traumatic arthritis, *ASA* American Society of Anesthesiologist.

**Table 2:** Preoperative data

**Parameter**	**≥80 years group mean (SD; range)**	**Control group mean (SD; range)**	***p*** **value**
KSS, points	38 (7.1; 18–56)	40 (4.8; 30–52)	0.245
KSFS, points	42 (8.1; 20–60)	44 (5.0; 30–56)	0.645
WOMAC, points	76.2 (9.1; 54–88)	75.4 (7.8; 53–83)	0.557
TFM	11.9° varus (20.3° valgus to 29° varus)	9.4° varus (8° valgus to 29.8° varus)	0.936
ROM	107° (20.9°; 35°–135°)	113° (12.8°; 70°–135°)	0.057

*SD* standard deviation, *KSS* Knee Society Score, *KSFS* Knee Society Function Score, *WOMAC* Western Ontario and McMaster Universities Osteoarthritis Index, *TFM* tibiofemoral mechanical axis, *ROM* range of knee motion.

**Table 3:** Prevalence of comorbidities

**Comorbidity**	**≥80 years group**	**Control group**	***p*** **value**
DM	19	20	0.973
Cardiac			
Hypertension	52	53	0.82
CAD	7	5	0.497
CHF	2	1	0.614
Arrhythmia	6	3	0.32
Liver cirrhosis	0	2	0.497
Renal insufficiency	4	4	0.951
Gout	2	3	0.684
COPD	5	7	0.598
Adrenocortical insufficiency	7	4	0.311
Peptic ulcer	13	11	0.575
Coagulopathy	0	2	0.497
Anemia^a^	17	14	0.459
Hemoglobin (g/dL), mean (SD; range)	12.7 (1.7; 8.8 ~ 16.7)	13.2 (1.4; 9.8 ~ 16.9)	0.115
Hematocrit, mean (SD; range)	39.1 (4.0; 30.5 ~ 48.1)	40.2 (3.9; 30.3 ~ 49.9)	0.082

*DM* diabetes mellitus, *CAD* coronary artery disease, *CHF* congestive heart failure, *CVA* cerebral vascular accident, *COPD* chronic obstructive pulmonary disease, *SD* standard deviation. ^a^Hb < 12 g/dL.

At the latest follow-up, compared to the preoperative scores, both groups had improved in the KSS, KSFS, and WOMAC scores (*p* < 0.001).

The tube drainage amount, wound length, length of stay, mean postoperative KSS, KSFS, WOMAC score, ROM, and TFM of the knee were all similar between two groups (Table 4).

**Table 4:** Intraoperative and postoperative data

**Parameter**	**≥80 years group mean (SD; range)**	**Control group mean (SD; range)**	***p*** **value**
Tube drainage, mL	493 (173; 201–1,015)	460 (179; 188–920)	0.429
Wound length, cm	10.3 (1.1; 7.8–13)	9.9 (0.9; 8–13)	0.107
Length of stay, day	6.1 (1.3; 5–11)	5.7 (1.2; 4–12)	0.061
KSS, points	86 (4.1; 72–92)	88 (4.1; 76–94)	0.77
KSFS, points	87 (3.8; 70–88)	89 (4.0; 78–92)	0.936
WOMAC, points	15.0 (7.9; 2–39)	14.6 (6.4; 1–35)	0.681
TFM	0.22° valgus (2.9° valgus to 2.4° varus)	0.05° varus (2.4° valgus to 2.6° varus)	0.109
ROM	118° (8.7°; 95°–145°)	120° (11.4°; 90°–140°)	0.811

*KSS* Knee Society Score, *KSFS* Knee Society Function Score, *WOMAC* Western Ontario and McMaster Universities Osteoarthritis Index, *TFM* tibiofemoral mechanical axis, *ROM* range of knee motion.

As for postoperative complications, the ≥80 years group had a higher rate of confusion (6.7% versus 0%, *p* = 0.026) and a higher rate of blood transfusion (29.3% versus 10.7%, *p* = 0.003) than controls. After Bonferroni correction, only blood transfusion reached significant difference. Two patients in the ≥80 years group and one control developed pneumonia after surgery. One patient in the ≥80 years group needed intensive care because of respiratory failure related to pneumonia. One control had acute myocardial infarction after surgery and underwent cardiac catheterization and stenting for coronary artery stenosis. One patient in the ≥80 years group and one control developed distal DVT after surgery and were treated with low-molecular-weight heparin followed by oral vitamin-K antagonist for 3 months. One control developed distal DVT 6 weeks after surgery and treated with oral vitamin-K antagonist only for 3 months. One control with a history of PE developed recurrent PE 4 days after surgery and was transferred to the cardiology department for further treatment. One patient in the ≥80 years group had postoperative CHF because of fluid overload. Three patients in the ≥80 years group had UGI bleeding. Two of them used indomethacin by suppository and did not receive gastroprotective agents. The other one who had peptic ulcer history developed UGI bleeding 2 days after surgery. Three patients in the ≥80 years group and one control had atelectasis postoperatively and recovered after aggressive pulmonary therapy. Other postoperative complications were similar between two groups (Table 5). One octogenarian patient had a periprosthetic fracture of the distal femur after TKA and was treated with open reduction and internal fixation with a blade plate and bone grafts. No patient in either group sustained deep infection or loosening of the prosthesis. There was no 90-day mortality in either group but one patient in the ≥80 years group died from unrelated causes more than 1 year after operation.

**Table 5:** Number of complications within 90 days after total knee arthroplasty

**Complication**	**≥80 years group**	**Control group**	***p*** **value**
Confusion, *n* (%)	5 (6.7%)	0 (0%)	0.026
Pneumonia, *n* (%)	2 (2.7%)	1 (1.3%)	0.614
MI, *n* (%)	0 (0%)	1 (1.3%)	0.243
DVT with positive venogram, *n* (%)	1 (1.3%)	2 (2.7%)	0.584
ICU care, *n* (%)	1 (1.3%)	1 (1.3%)	0.976
Postop CHF, *n* (%)	1 (1.3%)	0 (0%)	0.49
PE, *n* (%)	0 (0%)	1 (1.3%)	0.326
UGI bleeding, *n* (%)	3 (4%)	0 (0%)	0.115
Anemia, *n* (%)	17 (22.7%)	18 (24%)	0.959
Atelectasis, *n* (%)	3 (4%)	1 (1.3%)	0.359
Superficial wound infection, *n* (%)	4 (5.3%)	1 (1.3%)	0.203
Number of patients receiving transfusion, *n* (%)	22 (29.3%)	8 (10.7%)	0.003*

*MI* myocardial infarction, *DVT* deep vein thrombosis, *CHF* congestive heart failure, *PE* pulmonary embolism, *UGI* upper gastrointestinal, *ICU* intensive care unit. *Bonferroni-adjusted significance level of 0.00416.

## Discussion

Our study demonstrated that the both groups had improved clinical outcomes and patient satisfaction after TKA. In a long-term-follow-up study of 100 patients who were aged ≥80 years and underwent total joint arthroplasty (70 hips, 30 knees), the satisfaction rate was high (95%), and 97% of patients maintained a degree of independent living [12]. In our study, the mean postoperative KSS and KSFS in the ≥80 years group were 86 and 87 points, respectively, similar to the mean postoperative scores in the control group (88 and 89 points, *p* = 0.898 and *p* = 0.936, respectively). With regard to satisfaction, the mean postoperative WOMAC score was also similar between the two groups (15.0 versus 14.6, *p* = 0.817), indicating both groups of patients were satisfied with TKA. As for the radiographic results, there were no outliers (>3° deviation from the ideal, 0°) in either group. These results may explain the high patient satisfaction rate in our series.

It has been reported that the 90-day mortality rate after primary standard TKA was 0.46% [13]. Risk factors that increased the mortality after TKA included age >70 years; use of a cemented prosthesis; preexisting cardiopulmonary disease; and simultaneous bilateral arthroplasty [14]. The ≥80 years patient population had a higher risk of prevalence of comorbidities, including coronary disease and impaired myocardial function, which were related to postoperative complications and increased mortality [4]. Surgical volume is also related to short-term mortality after TKA. Lavernia and Guzman reported a significantly higher mortality rate in patients of low-volume surgeons (<10 per year) than medium- (10–100 per year) and high-volume (>100 per year) surgeons [15]. Kreder et al. [16] reported that, compared with patients aged 65 to 79 years, octogenarians carried a 3.4-time greater risk of mortality, a 3.5-time greater risk of pneumonia, and a 2.7-time greater risk of myocardial infarction after hip or knee arthroplasty. The TKA mortality rate was 1.09% in the octogenarians and 0.32% in the younger group. Our study did not find a higher 90-day mortality rate in the ≥80 years group: there were no deaths within 90 days in either group. We attribute this to the following: first, our patient sample was small; second, we selected patients with low risk through a preoperative consultation of cardiologist and neurologist; and third, we belong to a group of high-volume (>100 per year) arthroplasty surgeons.

In this study, we found a higher rate of postoperative confusion (6.7% versus 0%, *p* = 0.026) in the ≥80 years group compared with the control group, although it was not significant after Bonferroni correction. Kreder et al. [16] reported postoperative confusion was significantly higher in the octogenarian (2.33% vs 0.68%, with an OR of 3.6). The risk factors related to postoperative confusion are advanced age and general anesthesia [17]. Most of our patients (97.3%) underwent general anesthesia (Table 1), which may contribute to the higher rate of postoperative confusion among octogenarians.

Transfusion rate was greater among the ≥80 years group than the control group (29.3% versus 10.7%, *p* = 0003). Clement et al. [3] also reported a higher transfusion rate among octogenarians compared to the younger patients after standard TKA. However, the transfusion rate for their octogenarian patients was much lower (19.7%). We attribute this to the following reasons. First, our ≥80 years group had low mean preoperative levels of Hb and Hct, although they did not reach statistical significances (Table 3). Additionally, according to our hospital policy, a minimum Hb level of 10 g/dL is mandatory before any major operation, including TKA (4 patients aged ≥80 years and 1 younger patient had preoperative Hb ≤ 10 g/dL). Second, postoperative UGI bleeding was more frequent in the ≥80 years group than in the controls (4% versus 0%). This may have contributed to their higher rate of transfusion.

There was 4% rate (3 patients) of postoperative UGI bleeding in the ≥80 years group although it was not significantly different. Two patients used indomethacin by suppository post-operatively and did not receive gastroprotective agents. The other one had peptic ulcer history. After this study, we used gastroprotective agents such as proton pump inhibitors routinely in patients with history of peptic ulcer or in all patients who receive indomethacin for pain relief postoperatively.

We acknowledge the limitations of this study. It was a retrospective design, but we utilized a matched case-controlled study design to ensure that we accounted for potentially confounding risk factors as possible. It would be difficult or impossible to randomize the patients aged ≥80 years or older to a group that did or did not receive preoperative consultation. However, for the safety of elderly patients and to avoid unexpected postoperative complications, it has become our routine protocol for all patients aged ≥80 years and younger patients with history of cardiovascular or cerebrovascular events undergoing major orthopedic surgery.

## Conclusion

Our study showed that TKA was an effective procedure for patients aged 80 years or more. However, the ≥80 years group had a higher complication rate of blood transfusion compared with the younger patient group. With a preoperative evaluation by cardiologists and neurologists, patients aged 80 years or older have a low postoperative cardiovascular or cerebrovascular complications and 90-day mortality after TKA.

## Competing interests

The authors declare that they have no competing interests.

## Authors' contributions

FCK conceived of the study, participated in the study design, and drafted the manuscript. CHH performed the statistical analysis. WSC participated in the data acquisition and helped in drafting the manuscript. JWW performed the final approval of the version to be submitted. All authors read and approved the final manuscript.

## References

[B1] Bruyère O Ethgen O Neuprez A Zégels B Gillet P Huskin JP Reginster JY Health-related quality of life after total knee or hip replacement for osteoarthritis: a 7-year prospective study Arch Orthop Trauma Surg 2012 132 1583 1587 10.1007/s00402-012-1583-7 22842917

[B2] Keurentjes JC Van Tol FR Fiocco M Schoones JW Nelissen RG Minimal clinically important differences in health-related quality of life after total hip or knee replacement: a systematic review Bone Joint Res 2012 1 71 77 10.1302/2046-3758.15.2000065 23610674 PMC3626243

[B3] Clement ND MacDonald D Howie CR Biant LC The outcome of primary total hip and knee arthroplasty in patients aged 80 years or more J Bone Joint Surg Br 2011 93 1265 1270 10.1302/0301-620X.93B9.25962 21911540

[B4] Fleisher LA Beckman JA Brown KA Calkins H Chaikof EL Fleischmann KE Freeman WK Froehlich JB Kasper EK Kersten JR Riegel B Robb JF ACCF/AHA focused update on perioperative beta blockade incorporated into the ACC/AHA 2007 guidelines on perioperative cardiovascular evaluation and care for noncardiac surgery J Am Coll Cardiol 2009 2009 54 e13 e118 10.1016/j.jacc.2009.07.010 19926002

[B5] Blacker DJ Flemming KD Wizdicks EF Risk of ischemic stroke in patients with symptomatic vertebrobasilar stenosis undergoing surgical procedures Stroke 2003 34 2659 2963 10.1161/01.STR.0000092120.03676.D6 14526038

[B6] Blacker DJ Flemming KD Link MJ Brown RD Jr The preoperative cerebrovascular consultation: common cerebrovascular questions before general or cardiac surgery Mayo Clin Proc 2004 79 223 229 10.4065/79.2.223 14959917

[B7] Evans B Wijdicks E High-grade carotid stenosis detected before general surgery: is endarterectomy indicated? Neurology 2001 57 1328 1330 10.1212/WNL.57.7.1328 11591861

[B8] Haas SB Cook S Beksac B Minimally invasive total knee replacement through a mini midvastus approach: a comparative study Clin Orthop Relat Res 2004 428 68 73 10.1097/01.blo.0000147649.82883.ca 15534521

[B9] Pearse EO Caldwell BF Lockwood RJ Hollard J Early mobilisation after conventional knee replacement may reduce the risk of postoperative venous thromboembolism J Bone Joint Surg Br 2007 89 316 322 10.1302/0301-620X.89B3.18196 17356141

[B10] Wang CJ Wang JW Weng LH Prevention of deep-vein thrombosis after total knee artrhoplasty in Asia patients: comparison of low-molecular-weight heparin and indomethacin J Bone Joint Surg Am 2004 86-A 136 140 14711956 10.2106/00004623-200401000-00020

[B11] Perioperative red blood cell transfusion JAMA 1988 260 2700 2703 10.1001/jama.1988.03410180108040 3054179

[B12] Shah AK Celestin J Parks ML Levy RN Long-term results of total joint arthroplasty in elderly patients who are frail Clin Orthop Relat Res 2004 425 106 109 10.1097/00003086-200408000-00015 15292795

[B13] Gs G Mills D Joshi AB Mortality following primary total knee arthroplasty J Bone Joint Surg Am 2003 85-A 432 435 12637427 10.2106/00004623-200303000-00005

[B14] Parvizi J Sullivan TA Trousdale RT Thirty-day mortality after total knee arthroplasty J Bone Joint Surg Am 2001 83-A 1157 1161 11507123 10.2106/00004623-200108000-00004

[B15] Lavernia CJ Guzman JF Relationship of surgical volume to short-term mortality, morbidity and hospital charges in arthroplasty J Arthroplasty 1995 10 133 140 10.1016/S0883-5403(05)80119-6 7798093

[B16] Kreder HJ Berry GK McMurtry IA Halman SI Arthroplasty in the octogenarian: quantifying the risks J Arthroplasty 2005 20 289 293 10.1016/j.arth.2004.09.024 15809944

[B17] Ishii K Akiyama D Hara K Makita T Sumikawa K Influence of general anesthetics on the incidence of postoperative delirium in the elderly Masui 2011 60 856 858 21800669

